# P-2073. COVID-19 Vaccine Effectiveness in Patients with Lung Cancer and Mesothelioma in the Omicron Era

**DOI:** 10.1093/ofid/ofae631.2229

**Published:** 2025-01-29

**Authors:** Seyed M Hosseini-Moghaddam, Frances A Shepherd, Sarah Swayze, Jeffrey Kwong

**Affiliations:** University Health Network, Toronto, Ontario, Canada; University Health Network, Toronto, Ontario, Canada; ICES Ontario, Toronto, Ontario, Canada; ICES, Toronto, Ontario, Canada

## Abstract

**Background:**

Since the Omicron variant emerged, uptake of booster doses of COVID-19 vaccines has gradually decreased both in the general population and among individuals at increased risk of severe COVID-19, such as patients with lung cancer. COVID-19 vaccine effectiveness (VE) has not been estimated in patients with lung cancer in the Omicron era.

**Methods:**

In this test-negative design study using linked population-based cancer registry, health administrative, vaccination, and public health surveillance databases, we included all patients with active lung cancer or mesothelioma living in Ontario, Canada, who were tested for SARS-CoV-2 by RT-PCR from January 2, 2022, to August 31, 2023. We estimated VE against COVID-19-related severe outcomes (hospitalization or death) 7-179 days and ≥180 days following vaccination.

**Results:**

During the study period, 13,622 patients with active lung cancer or mesothelioma underwent SARS-CoV-2 testing, with 1,371 (10.1%) positive. After exclusions, we analyzed 6,037 testing episodes, including 1,354 test-positive and 4,683 randomly selected test-negative episodes. Overall, COVID-19-associated hospitalization and mortality rates were 7.3% and 4.0%, respectively. Across both groups, 126/439 hospitalized patients (28.7%) and 116/5,598 non-hospitalized patients died (2.1%) (p=0.001). After multivariable adjustment, VE against severe COVID-19 was 56% (95%CI, 29%, 72%) 7-179 after vaccination and 10% (-45%, 44%) ≥180 days after vaccination.

**Conclusion:**

Overall, COVID-19 VE against severe outcomes appears to be considerably lower for patients with active lung cancer or mesothelioma than the general population and decreases substantially beyond 180 days after vaccination. Our finding supports administration of COVID-19 booster doses in patients with lung cancer and mesothelioma every 6 months.

**Disclosures:**

All Authors: No reported disclosures

